# Cancer-Associated Venous Thromboembolism: A Practical Review Beyond Low-Molecular-Weight Heparins

**DOI:** 10.3389/fmed.2017.00142

**Published:** 2017-08-28

**Authors:** Alannah Smrke, Peter L. Gross

**Affiliations:** ^1^Department of Medicine, McMaster University, Hamilton, ON, Canada; ^2^Thrombosis and Atherosclerosis Research Institute, Hamilton, ON, Canada

**Keywords:** cancer-associated venous thromboembolism, cancer-associated thrombosis, venous thromboembolism, pulmonary embolism, low-molecular-weight heparin, direct oral anticoagulants

## Abstract

Patients with cancer are at significantly higher risk of developing, and dying from, venous thromboembolism (VTE). The CLOT trial demonstrated superiority of low-molecular-weight heparins (LMWH) over warfarin for recurrent VTE and established LMWH as the standard of care for cancer-associated VTE. However, with patients living longer with metastatic cancer, long-term injections are associated with significant cost and injection fatigue. Direct oral anticoagulants (DOACs) are an attractive alternative for treatment of cancer-associated VTE. Meta-analysis of subgroup data of patients with cancer from the large DOAC VTE trials and small non-randomized studies have found no difference in VTE recurrence or major bleeding. With this limited evidence, clinicians may decide to switch their patients who require long-term anticoagulation from LMWH to a DOAC. This requires careful consideration of the interplay between the patient’s cancer and treatment course, with their underlying comorbidities.

## Introduction

Patients with cancer are at a four-fold higher risk (1) of developing venous thromboembolism (VTE), which includes pulmonary embolism and deep vein thrombosis and have three-fold higher mortality compared to matched peers without VTE (2). The current standard of care is low-molecular-weight heparin (LMWH), although some guidelines permit use of vitamin K antagonists (VKAs) (3). In patients with metastatic disease, anticoagulation often continues indefinitely. However, adherence to treatment guidelines recommending LMWH is far from ideal with 30–60% of patients in different cohorts not on LMWH, in the era before direct oral anticoagulants (DOACs) (4). In early trials, 8% of patients declined participation because of needles (5) and 17% were not “satisfied or very satisfied” with needle administration (6). This injection fatigue contributes to non-compliance (7). Also, long-term use of LMWH is associated with significant cost. For non-cancer patients, DOACs are recommended over VKAs for VTE treatment. When given a hypothetical choice of LMWH versus an oral agent with equal efficacy, most patients with cancer chose the oral agent (8). Despite the lack of direct evidence supporting DOACs as a non-inferior choice to LMWH to treat cancer-associated VTE, current practice patterns suggest that as many as 25% of such patients are being treated with DOACs and 30% are being treated with VKAs (9). The most recent ACCP guideline stated that when LMWH is not used in cancer-associated VTE, there is no preference for VKAs over DOACs (3). Here, we will review the evidence and considerations for use of DOACs in cancer-associated VTE.

## Evidence Summary (Table 1)

### Low-Molecular-Weight Heparins

The CANTHANOX randomized clinical trial (RCT) of 146 patients compared enoxaparin versus warfarin for 3 months in France. At 6 months, more patients with cancer-associated VTE treated with warfarin (21.1%) compared to LMWH (10.5%) reached the primary outcome of major bleeding or recurrent VTE. The difference was mainly driven by major bleeding events (10).

**Table 1:** Summary of randomized data for anticoagulant treatment of cancer-associated venous thromboembolism (VTE) (37).

	Trial	Anticoagulant	Recurrent VTE		Major bleeding	
	
			*N*	%	*N*	%
Randomized controlled trial	ONCENOX (12)	Enoxaparin bridging to warfarin	3/30	10	1/34	2.9
		Enoxaparin^a^	2/29	6.9	2/31	6.5
		Enoxaparin^b^	2/32	6.3	4/36	11.1
	LITE (15, 16, 32, 33)	Usual care (IV heparin or warfarin)	16/100	16*	7/100	7
		Tinzaparin (175 U/kg/day)	7/100	7*	7/100	7
	CATHENOX (38)	Warfarin	3/75	6.7^c^	12/75	16
		Enoxaparin (1.5 mg/kg/day)	2/71	2.8	5/71	7.0
	CLOT (13)	Warfarin	53/336	15.8*	12/335	3.6
		Dalteparin	27/336	8.0*	19/338	5.6
	CATCH (10)	Warfarin	45/451	10.5	11/451	2.4
		Tinzaparin	31/449	7.2	12/449	2.1
Subgroup analysis	AMPLIFY (11)	Warfarin	5/78	6.4	4/80	5.0
		Apixaban	3/81	3.7	2/87	2.3
	Hokusai-VTE (37)	Warfarin	7/99	7.1	3/99	3
		Edoxaban	4/109	3.7	5/109	4.6
	EINSTEIN (PE + DVT) (33)	Warfarin	8/204	3.9	8/204	3.9
		Rivaroxaban	6/258	2.3	5/238	2.1
	RECOVER (I, II) (32)	Warfarin	3/57	5.3	3/57	5.3
		Dabigatran	2/64	3.1	6/159	7.8

* = *p* < 0.05.

*^a^1.0 mg/kg/q12h for 5 days, then 1.0 mg/kg/day*.

*^b^1.5 mg/kg/day*.

*^c^Composite end point of major bleeding, VTE, or death*.

The randomized comparison of LMWH versus oral anticoagulant therapy for the prevention of recurrent VET in patients with cancer (CLOT) trial (11) established LMWH as the standard of care for treatment of cancer-associated VTE. This multicenter trial randomized 676 patients with cancer, defined as those diagnosed within cancer 6 months prior to enrollment [excluding basal (BCC) and squamous cell carcinoma (SCC) of the skin], or recurrent or metastatic disease, to dalteparin or warfarin. Dalteparin was superior to warfarin in terms of risk of recurrent thromboembolism (9% versus 17%, *p* = 0.002) with no significant difference in bleeding or mortality.

Subsequently, two small studies comparing warfarin and LMWH, ONCENOX (12) and LITE (10), demonstrated no difference in major or minor bleeding, or major bleeding and mortality, respectively, between warfarin and LMWH. Importantly, the single-arm DALTECAN study found that extending treatment duration to up to 12 months of LMWH resulted in no increase in the annualized rates of recurrent VTE or major bleeding (10).

A more recent evaluation of the benefit of LMWH over VKAs is the Comparison of Acute Treatments in Cancer Hemostasis (CATCH) trial (13), a randomized multinational study that compared the LMWH tinzaparin to warfarin in 900 patients with cancer. Active cancer was defined as pathologically confirmed malignancy (excluding BCC and SCC) plus any of the following: diagnosis within 6 months, recurrent, locally advanced or metastatic disease, treatment for cancer within previous 6 months, and non-complete remission from hematological malignancy. There was no significant difference (7.2% versus 10.5%, *p* = 0.07) between tinzaparin and warfarin for recurrent VTE.

### Direct Oral Anticoagulants

There are currently no published randomized trials using DOACs exclusively for cancer-associated VTE. We will focus our review on DOACs which are approved for VTE treatment, the direct factor Xa inhibitors apixaban, edoxaban, dabigatran, and rivaroxaban. Evidence for treating cancer-associated VTE with DOACs can be extrapolated from subgroup analysis of patients with cancer in large trials of VTE (14). The criterion for active cancer is vaguely detailed in these trials, though patients with aggressive cancer or to be treated with LMWH were excluded.

Three published meta-analyses (15) of cancer patients treated with DOACs versus warfarin found no significant difference between recurrent VTE and major bleeding.

A recent small prospective study of 200 patients (16) and retrospective review of 237 patients with active cancer (47% had metastatic disease) (17–19) and treated with rivaroxaban found low rates of recurrent VTE (4.4, 3.8%, respectively) and major bleeding (1.3, 2.2%).

### Ongoing Studies

An RCT of edoxaban versus dalteparin (Hokusai-Cancer) has a target completion date of December 2017 (20). In this trial, eligible patients were those with cancer diagnosed within 2 years prior to enrollment or active cancer (other than BCC or SCC of the skin) defined as one of the following: diagnosed within 6 months or recurrent, regionally advanced or metastatic or receiving treatment 6 months prior to randomization, or hematologic malignancy not in complete remission. In this open-label trial, after 7 days of dalteparin, patients are randomized to dalteparin or edoxaban. Primary outcomes are recurrent VTE and major bleeding. The trial did not include a protocol for management of thrombocytopenia in the DOAC arm. A reduced dose of edoxaban was given to patients while they received certain P-glycoprotein inhibitors.

Additionally, a single-site RCT comparing rivaroxaban to LMWH (tinzaparin, dalteparin, or enoxaparin) in patients with active malignancy, Karnofsky Performance Status >70% has a target completion date of March 2018 (21). The primary outcome is patient satisfaction, with secondary outcomes including recurrent VTE, major bleeding, and patient compliance. Finally, an RCT only enrolling in the United States comparing apixaban to dalteparin with primary outcomes of major bleeding and secondary outcomes including recurrent VTE is expected to be completed in November 2018 (22).

There are also ongoing registries, such as COSIMO (23) and ISRCTN Registry 86712308 (select-d) (24), which will inform the role of DOACs in this population.

## Discussion

The results of the CLOT trial changed the standard of care for cancer-associated VTE by offering a reduction of recurrent VTE with LMWH, but a similar bleeding risk compared to warfarin. However, these results were not replicated in CATCH. Importantly, CATCH enrolled a lower risk cancer population. For example, a woman theoretically cured with lumpectomy and radiation for breast cancer would have been eligible for enrollment in CATCH, but not CLOT. The all-cause mortality in CATCH was lower (30.6–33.6%), compared to CLOT (39–41%). Thus, CLOT enrolled an overall sicker, more symptomatic cancer population with likely a higher risk of recurrent VTE. This population would have more difficulty with consistent oral intake due to disease or treatment-related nausea, vomiting, and anorexia, predisposing them to difficult titration of warfarin. LMWHs in this population provide reliable anticoagulation independent of diet or drug interactions.

Thus, to compare the results across trials, the risk profile of the cancer population must be scrutinized. The lower rates of recurrent VTE in subgroups of cancer patients within the large DOAC VTE trials suggest that, overall, they were a low-risk population.

In patients with metastatic disease or at high risk of recurrence, the standard of care is indefinite anticoagulation (25). This poses a great financial burden to patients and the health care system – in the United States and Ontario, Canada per month, respectively, LMWH costs approximately ($US, $CAD) 3,500, 960–1,200, DOACs $350–400, 86–96, and warfarin $5–10, $2–5 (26, 27).

Ongoing trials comparing DOACs to LMWH may not provide a clear answer. The Hokusai-cancer trial, recruited 1,000 patients, is closed to recruitment and follow-up is ongoing. If this RCT shows that edoxaban is inferior to LMWH then this will curtail the use of DOACs in cancer-associated VTE. If, however, Hokusai shows that edoxaban is non-inferior to dalteparin, then open questions will remain. Will this result be generalizable to all direct factor Xa inhibitors? Whether an all-oral regimen will be acceptable to treat acute VTE may be answered by the apixaban and rivaroxaban trials (23, 24). However, these are single center or country trials that may have limited generalizability. Hokusai does not have a formal protocol for management of thrombocytopenia, which is a concern for anticoagulant use in cancer patients; how should DOACs be managed in the context of thrombocytopenia? The dose of edoxaban was lowered when patients took certain P-glycoprotein inhibitors; is this transferable to other direct factor Xa inhibitors?

## DOACs in Practice

Given their cost and ease of administration, DOACs are an attractive alternative to LMWH. Based on probable equivalence data from non-cancer VTE subgroup analyses, in practice some patients with cancer-associated VTE are being switched to DOACs (Table 2). Navigating this switch presents a new challenge to clinicians.

**Table 2:** Patients who can be considered for a switch to direct oral anticoagulants.

Probably	Probably not
Stable metastatic cancer on oral or maintenance intravenous treatment Metastatic cancer stable post immunotherapy treatment Metastatic malignancy with expected long survival (ER + breast cancer on oral agent, low-grade lymphoma)	Metastatic cancer on immunotherapy treatment Metastatic cancer progressing on treatment Metastatic malignancy with expected short survival (pancreatic cancer, esophageal cancer, and refractory high-grade lymphoma)

## Acute Cancer-Associated VTE

Low-molecular-weight heparin remains standard of care for management of acute VTE in cancer patients. Guidelines currently recommend at least 6 months of therapy with LMWH upfront. Recent ACCP 2016 guidelines do not have a preference for VKA versus DOAC for patients with cancer-associated VTE who are not treated with LMWH (3).

## After Acute Treatment of Cancer-Associated VTE

**Figure 1:**
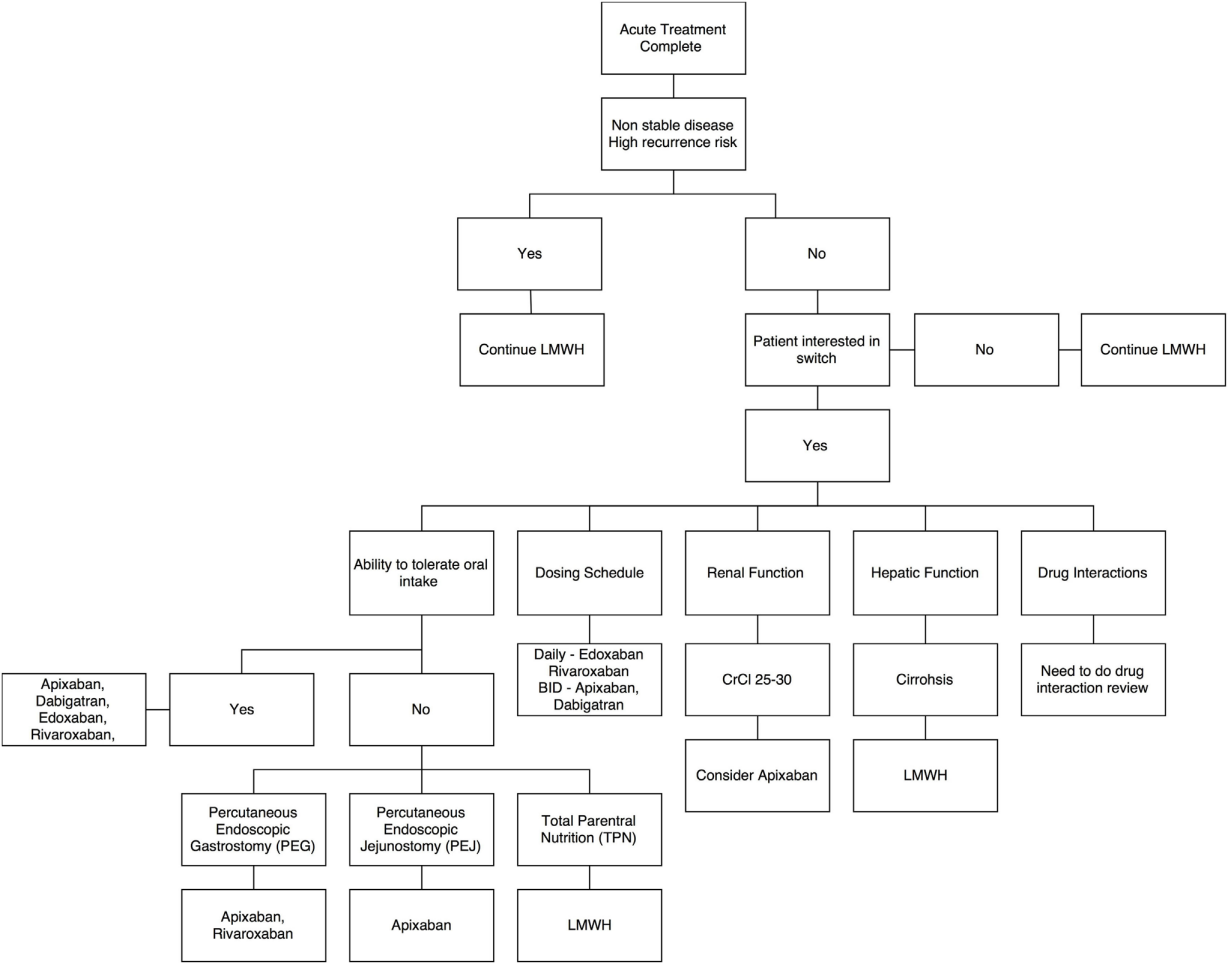
Decision aid for switch from low-molecular-weight heparins to direct oral anticoagulants – a summary algorithm.

With limited evidence, there is an emerging role for DOACs in the long-term management of cancer-associated VTE. Patients who are felt to be at high risk for recurrent VTE would likely benefit from continuing LMWH. Beyond high-risk patients, clinicians may consider individual patient factors to recommend a treatment plan (Figure 1).

### Treatment-Related Considerations

Risk of VTE is six-fold higher on intravenous chemotherapy (23, 24). For this reason, LMWH is continued during treatment with intravenous chemotherapy. Thus, a switch can be considered once the patient is on an oral chemotherapy agent or on a treatment break. These patients are considered to have a relatively lower risk of recurrent VTE and bleeding.

Patients with stable metastatic disease maintained on intravenous chemotherapy – for example, anti-Her2 therapy for breast cancer – can be considered for switch to a DOAC. The safety of anticoagulants with bevacizumab and related agents needs study.

### Disease-Related Considerations

High-risk patients who should continue on LMWH would include rapidly progressing disease on or off treatment.

### Oral Intake

Rivaroxaban must be taken with food (3) and requires administration within the stomach (PEG tube) to ensure consistent bioavailability (28, 29). Apixaban can be given independent of food intake *via* PEG/PEJ tubes (30). There are limited data for edoxaban, and currently it is only recommended as an intact tablet (31). The bioavailability of dabigatran is significantly increased when removed from its capsule, therefore, is not recommended to be taken *via* PEG/PEJ (31).

### Renal Function

All the non-cancer DOAC trials (31) excluded patients with creatinine clearance (CrCl) less than 30, except AMPILFY, who excluded if CrCl < 25 mL/min; and most patients had CrCl of over 50 mL/min. Thus, patients with a CrCl above 30 mL/min are a candidate for all DOACs. With this limitation, apixaban may be useful for patients with a CrCl between 25 and 30 mL/min; however, clinicians must carefully discuss the use of a DOAC with patients who have this level of renal dysfunction.

### Hepatic Function

The DOAC VTE trials generally excluded patients with significant liver disease. Though not clearly defined, laboratory exclusions were AST/ALT greater than 2 times, or bilirubin greater than 1.5 times the upper limit of normal, respectively (30).

If a patient’s malignancy is the major contribution to the liver dysfunction (i.e., majority of the liver is replaced with tumor), this likely overall tends a poor prognosis, as many of the chemotherapy regimens require good hepatic function to safely administer. Unless the cancer is indolent, and synthetic function is preserved (i.e., low grade neuroendocrine tumor), patients with significant hepatic involvement with cancer-associated VTE would be best treated with LMWH.

### Thrombocytopenia

Clinicians have more experience using LMWH with thrombocytopenia than DOACs. LMWH is often given at full dose when the platelet count is >50 × 10^9^/L, although this has not been validated in prospective studies (15, 16, 32). Only AMPLIFY specified inclusion criteria of a platelet count >100 × 10^9^/L (15, 16, 32, 33). In practice, a platelet count greater than 100 × 10^9^/L is generally required to be a candidate for a DOAC. Evidence of DOAC safety with lower platelet counts is lacking.

### Drug Interactions

Direct oral anticoagulants rely on P-glycoprotein and CYP3A4 for metabolism, so drugs that alter (induce or suppress) both of these metabolic pathways should be avoided (Table 3) (34). This mandates a comprehensive drug evaluation, especially for patients with borderline CrCl. It is generally accepted that drugs that are metabolized by these pathways, without inducing or suppressing them, are not a concern.

**Table 3:** Common modulators of P-glycoprotein and CYP3A4 function (33).

	Inhibitors	Inducers
P glycoprotein	*Cyclosporine, Tacrolimus, Tamoxifen*, diltiazem, verapamil, progesterone, and amiodarone	St John’s Wort, paclitaxel, phenytoin, and rifampin
CYP3A4	Cytarabine, *imatinib*, ketoconazole, tamoxifen, anastrozole, and grapefruit juice	St John’s Wort, corticosteroids, carbamazepine, phenobarbital, and phenytoin

*The underscored are drugs contraindicated in Hokusai-cancer VTE trial, while those in italics resulted in a dose reduction of the edoxaban (partial listing)*.

### Anticoagulant potency

In practice the dose of LMWH can be titrated, either, in mild thrombocytopenia, or to alleviate minor bleeding. This practice will be difficult to extrapolate to DOACs where there are less options for lower doses.

### Palliative Care

There are no published data surrounding the use of DOACs at the end of life. VTE at the end life results in significant morbidity and is a concern for patients (35, 36). A qualitative study of patients with metastatic cancer not receiving active treatment found that patients found LMWH was an acceptable, necessary inconvenience to prevent VTE (35, 36). Clinicians can consider an informed switch with patients receiving symptom management who can tolerate oral intake to reduce the risk of thrombosis, but want to avoid injections.

## Managing the Patient on a DOAC – Review of Cases

An important consideration of a patient on a DOAC is management of complications, most importantly bleeding and recurrent VTE.

### Case 1

Mrs. A is a 68-year old female with metastatic lung cancer with a symptomatic PE, initially treated with LMWH for 12 months, then was switched to a DOAC when her cancer was stable and she was on a chemotherapy holiday. She presents with a recurrent symptomatic PE while on this DOAC.

### Case 1 – Recurrent VTE on a DOAC

Mrs. A had stable metastatic lung cancer on a treatment break and was switched to a DOAC after at least 6 months of LMWH. She developed a recurrent VTE on DOAC therapy, necessitating a switch to LMWH. Compliance should be assessed; a compliant patient would likely benefit from continuation of LMWH indefinitely.

### Case 2

Mr. S is a 56-year old male with metastatic melanoma with a symptomatic DVT. He was initially treated with LMWH for 6 months, then switched to a DOAC after he had completed immunotherapy and his imaging showed stable disease. He presents with a hemodynamically significant gastrointestinal bleed while on this DOAC.

### Case 2 – Hemodynamically Significant Bleeding on a DOAC

Mr. S had metastatic melanoma and a DVT which was initially treated with LMWH and then switched to a DOAC when his disease was stable.

The initial management of his gastrointestinal bleeding should be the same as if he did not have cancer, including appropriate reversal agents. Once stabilized, he would benefit from a switch back to LMWH, as the dose can be titrated to alleviate bleeding risk. Long term, the decision to switch back to a DOAC would require understanding of his long-term risk of recurrent bleeding (i.e., resolved ulcer versus angiodysplasia).

## Conclusion

Cancer-associated VTE is common. The standard of care, LMWH, is costly and can be inconvenient for patients, leading to poor compliance. There is emerging evidence that DOACs may be at least equivalent to warfarin for treatment of cancer-associated VTE. Though the standard of care for acute cancer-associated VTE remains LMWH, in the appropriately selected patient, there may be a role for switching to DOACs. Results of ongoing trials will better inform clinicians regarding the safety and efficacy of DOACs for treatment of acute VTE.

## Author Contributions

AS wrote the review. PG conceived the review. AS and PG edited the review equally.

## Conflict of Interest Statement

PG has received speaker honoraria from Bayer, BMS, Pfizer, and Leo Pharma; grants from Bayer, BMS, and Boehringer-Ingelheim and holds intellectual property on a method to detect DOACs. AS declares that the research was conducted in the absence of any commercial or financial relationships that could be construed as a potential conflict of interest.
